# The Traffic Light Protocol: Preventing the 90° ‘Point of No Return’ Through Risk-Stratified Spinal Surveillance in Children with Cerebral Palsy

**DOI:** 10.3390/jcm15093205

**Published:** 2026-04-22

**Authors:** Michał Latalski, Anna Danielewicz, Martin Repko, Athanasios I. Tsirikos, Tomasz Kotwicki, Tomasz Potaczek, Johanna Syvänen, Paweł Grabala, Wiktor Urbański, Martin Prýmek, Piotr Janusz, Barbara Jasiewicz, Matti Ahonen, Ilkka Helenius

**Affiliations:** 1 Children’s Orthopaedic Department, Medical University of Lublin, 20-093 Lublin, Poland; anna.danielewicz@gmail.com; 2 Department of Orthopaedic Surgery, Faculty of Medicine, Masaryk University, 625-00 Brno, Czech Republic; repko.martin@fnbrno.cz (M.R.); prymek.martin@fnbrno.cz (M.P.); 3 University Hospital Brno, 625-00 Brno, Czech Republic; 4 Scottish National Spine Deformity Center, Royal Hospital for Children and Young People, 50 Little France Crescent Edinburgh BioQuarter, Edinburgh EH 16 4TJ, UK; atsirikos@hotmail.com; 5 Department of Spine Disorders and Paediatric Orthopedics, Wiktor Dega Orthopedic Institute, Poznan University of Medical Sciences, 61-545 Poznań, Poland; kotwicki@ump.edu.pl (T.K.); mdpjanusz@gmail.com (P.J.); 6 Department of Orthopedics and Rehabilitation, Medical College, Jagiellonian University, 34-500 Zakopane, Poland; tomaszpotaczek@gmail.com (T.P.); basiajasiewicz@gmail.com (B.J.); 7 Department of Paediatric Orthopaedic Surgery, University of Turku and Turku University Hospital, 20520 Turku, Finland; johvuo@gmail.com; 8 Department of Neurosurgery, Polish-Mother’s Memorial Hospital Research Institute, Rzgowska 281/289, 93-338 Lodz, Poland; pgrab2015@gmail.com; 9 Department of Neurosurgery, Wroclaw Medical University, 50-556 Wroclaw, Poland; wiktor.urbanski@umw.edu.pl; 10 Department of Paediatric Orthopaedics, Helsinki New Children’s Hospital, 00280 Helsinki, Finland; matti.ahonen@hus.fi (M.A.); ilkka.helenius@helsinki.fi (I.H.); 11 Department of Orthopaedics and Traumatology, Helsinki University Hospital, University of Helsinki, 00290 Helsinki, Finland

**Keywords:** cerebral palsy, neuromuscular scoliosis, spinal surveillance, GMFCS, Delphi method, pediatric spine surgery, pelvic obliquity, sitting radiographs

## Abstract

**Background**: Cerebral palsy (CP) is the leading cause of permanent physical disability in children. Although hip surveillance is a global standard, spinal surveillance remains inconsistent, often leading to reactive rather than proactive management of neuromuscular scoliosis. This study aims to establish an international consensus on a risk-based spinal surveillance protocol. **Methods**: A three-round modified Delphi process was conducted in 2024 with 15 international pediatric spine surgeons, identified through purposive sampling. The process adhered to CREDES standards and focused on establishing standards for timing, frequency, and radiographic surveillance. Consensus thresholds were defined a priori as excellent (≥80%) and good (≥73%) agreement. **Results**: The panel reached excellent consensus (93%) on a “Traffic Light” system based on the Gross Motor Function Classification System (GMFCS) levels. Green Group (Walkers, GMFCS I–II): Clinical surveillance. Amber Group (Poor Walkers, GMFCS III, and asymmetric hemiplegic GMFCS I–II): Annual radiographs starting at ages 3–8. Red Group (Non-Walkers, GMFCS IV–V): Six-monthly radiographs starting at ages 3–5. There was 100% consensus on the mandatory use of sitting radiographs for non-ambulatory patients to prevent masking true pelvic decompensation. Critical referral triggers were identified as a Cobb angle >20°, pelvic obliquity ≥5°, or a progression rate ≥1° per month. **Conclusions**: The “Traffic Light” protocol helps identify the “window of opportunity” for intervention before reaching the 90° “point of no return,” where surgical risks increase nonlinearly. This proactive approach aims to reduce surgical complications and systemic delays in specialized care.

## 1. Introduction

Cerebral palsy (CP) remains the leading cause of permanent physical disability in childhood [1]. Although the primary neurological damage is non-progressive, the secondary musculoskeletal effects—particularly neuromuscular scoliosis—are dynamic [2]. Neuromuscular scoliosis in patients with CP is a distinct clinical entity from adolescent idiopathic scoliosis (AIS). While AIS typically ceases to progress rapidly after skeletal maturity, CP-related scoliosis is driven by persistent, lifelong factors, including asymmetric muscle spasticity, truncal hypotonia, and poor postural control [3]. Unlike idiopathic scoliosis, neuromuscular curves in this group tend to begin early, progress rapidly, and involve the pelvis extensively [4]. Consequently, curve progression in CP often continues well into adulthood. In children classified at Gross Motor Function Classification System (GMFCS) levels IV and V, the prevalence of spinal deformity exceeds 90% [5,6]. This progressive spinal collapse often leads to loss of sitting balance and severe pulmonary problems, which directly increase the risk of early death and significantly reduce overall quality of life [7].

Despite the predictable and severe natural history of neuromuscular spinal deformities, international management strategies remain surprisingly fragmented. This stands in stark contrast to the management of hip displacement in CP. Over the past two decades, national and international hip surveillance programs, such as the widely successful CPUP registry originating in Sweden, have become a global standard of care [8]. These protocols have shifted the focus from salvage surgery to preventive preservation, demonstrating that regular, standardized, risk-stratified radiographic screening drastically reduces the incidence of severe joint contractures. However, a glaring clinical paradox remains: while a child with CP might have their hips meticulously monitored every 6 to 12 months according to strict guidelines, their spine—subject to the same neuromuscular forces—is often evaluated erratically, subjectively, or only when a gross clinical deformity becomes apparent to caregivers. No equivalent consensus-based framework exists for the spine, resulting in a “reactive” rather than “proactive” clinical approach.

This discrepancy highlights an urgent need for a paradigm shift in pediatric orthopedic care. Recent evidence underscores the severe risk of delayed referral, identifying a critical “point of no return” at a Cobb angle of about 90° [9]. Beyond this point, surgical risks increase nonlinearly, with infection rates rising threefold and the need for high-risk combined anterior–posterior surgeries increasing sixfold [9]. Furthermore, systemic surgical delays exceeding 8.5 months result in clinically meaningful curve progression averaging over 13° [10]. Missing the “window of opportunity” for safe intervention is compounded by systemic barriers in the referral process. Significant knowledge gaps among primary care pediatricians, limited parental awareness of the risk of rapid curve progression and the optimal timing for surgical referral, and geographic disparities in access to specialists often lead to patients presenting only after deformities have reached a rigid, life-threatening stage [11,12]. Additionally, the lack of standardized imaging protocols leads to inconsistent measurements; supine radiographs can underestimate coronal curves by up to 16° and obscure critical pelvic obliquity, an early sign of spinal decompensation [13,14].

To address these challenges, we conducted a three-round international Delphi process to develop a risk-stratified surveillance protocol. By implementing a “Traffic Light” system based on functional GMFCS levels, this study aims to provide clinicians with a practical, evidence-based guideline for early detection and management of neuromuscular scoliosis, ultimately shifting the focus from reactive salvage to proactive, anticipatory care.

## 2. Materials and Methods

### 2.1. Study Design and Chronology

The Delphi method was chosen as the primary methodology for this study because there is a significant lack of high-quality prospective evidence or randomized controlled trials on spine surveillance in CP. When empirical evidence is limited yet clinical decisions are still needed, the Delphi technique is a reliable scientific tool for distilling expert opinions into consensus for clinical practice [15]. The iterative process enables the systematic collection of anonymous judgments, which helps reduce cognitive biases, hierarchical influences, and the dominance of certain personalities that often skew results in traditional face-to-face expert panels [16].

Building on these principles, we used a modified Delphi technique to achieve expert consensus on spinal surveillance. The Delphi process was conducted throughout 2024 (Round 1: February–March; Round 2: May–June; Round 3: September–October). Between rounds, panelists received the percentage distribution of responses and an anonymized summary of qualitative comments to promote convergence. This multi-round approach allowed participants to consider the group’s responses and gradually refine their positions. The methodology strictly aligns with the Conducting and Reporting Delphi Studies (CREDES) checklist [17]. Notably, our independently established consensus thresholds closely align with the methodological framework published in 2025 by the European Pediatric Orthopedic Society (EPOS) spine study group [18], thereby providing retrospective validation of our approach against the current gold standard in European pediatric spinal research.

### 2.2. Panel Selection and Steering Committee

The process was overseen by a steering committee of three senior pediatric spine surgeons. The expert panel comprised 15 international surgeons. Selecting exactly 15 experts aligns with established methodological recommendations for Delphi studies in healthcare, which indicate that panels of 10 to 18 experts are optimal for achieving reliable consensus without causing data saturation or reducing response rates [15]. Participants were selected through purposive sampling from experts with highly specialized, high-volume clinical practice in neuromuscular deformity care within academic settings with access to advanced technologies. Minimum inclusion criteria required at least 10 years of clinical practice, a specific focus on pediatric neuromuscular spine deformities, and the annual evaluation of more than 50 CP patients. This sampling method ensured accurate evaluation of radiographic parameters and surgical thresholds. Anonymity was strictly preserved across all rounds using a secure, web-based application, ensuring that technical opinions carried equal weight and minimizing potential peer-group bias.

### 2.3. The Three-Round Delphi Process

The consensus-building process was designed to gradually focus on specific clinical variables.

Round 1 (Qualitative): Open-ended questions identified key variables related to the timing and frequency of surveillance. Thematic analysis of these responses [14] informed the development of 28 preliminary statements.

Round 2 (Quantitative): These statements were expanded into a detailed matrix of 135 specific items that assess exact age ranges, surveillance frequencies, and curve-magnitude thresholds for each GMFCS level. Statements were rated on a 5-point Likert scale, ranging from “strongly agree” to “strongly disagree”.

Round 3 (Convergence): Items that did not reach initial consensus were refined and compiled into 35 revised statements. To achieve agreement, these non-consensus items were reevaluated using a 3-point scale (agree, neutral, disagree). Participants received a summary of the group’s previous responses to guide their answers.

### 2.4. Data Management and Consensus Classification

Data were collected using secure, web-based tools to ensure an audit trail and data integrity. Because the completion rate was 100% among the 15 retained experts across all critical rounds, no missing data handling was necessary. Consensus thresholds were established beforehand.

Excellent Consensus: ≥80% agreement.

Good Consensus: ≥73% to 79% agreement.

For the final “Traffic Light” protocol, a minimum threshold of 73% (Good Consensus) was required to include a statement. Inter-rater reliability during the thematic analysis phase was high (κ = 0.87), and any coding discrepancies were resolved through consensus discussion with a third senior author.

**Figure 1:**
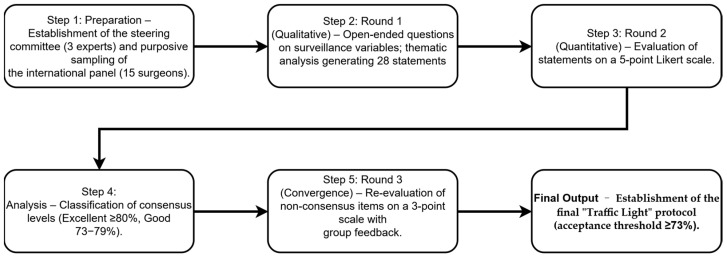
Flowchart of the modified Delphi study methodology.

The iterative Delphi process ensured the anonymous and systematic collection of expert opinions. Beginning with qualitative inquiry and progressing through rigorous quantitative rounds, the process concluded once a pre-established threshold for good-to-excellent consensus was reached for the clinical protocol (Figure 1).

## 3. Results

### 3.1. Expert Participation and Delphi Flow

Of the 15 invited international experts, 14 (93%) completed all three rounds of the Delphi process, indicating strong sustained engagement. The initial open-ended inquiry in Round 1 generated 28 preliminary statements on risk stratification, imaging standards, and referral triggers for neuromuscular scoliosis. In the subsequent quantitative rounds (Rounds 2 and 3), these statements were rigorously evaluated, refined, and consolidated into the final consensus protocol. The evolution of selected Delphi statements and voting outcomes across the rounds is summarized in Table S1, while the qualitative dataset from Round 1 is presented in Supplementary Table S2, and the detailed quantitative raw data from the expert consensus are available in Supplementary Data S2.

### 3.2. The “Traffic Light” Stratification System

The panel reached an excellent consensus (93%) on implementing a risk-stratified “Traffic Light” system to guide the intensity of surveillance (Table 1). Recognizing that the natural history of neuromuscular spinal deformity is closely linked to motor function, patients were divided into three pathways based on their GMFCS levels.

Green Group (Walkers, GMFCS I–II): Includes patients at the lowest risk of progressive structural deformity, for whom routine clinical assessment is considered adequate.

Amber Group (Poor Walkers, GMFCS III, and asymmetric hemiplegic GMFCS I–II): The panel explicitly included ambulant patients with hemiplegia in this high-risk group. In this subgroup, unilateral spasticity and muscle tone imbalance often lead to marked clinical trunk asymmetry and pelvic obliquity, indicating a markedly increased risk of secondary structural deformity that requires systematic surveillance.

Red Group (Non-Walkers, GMFCS IV–V): This is the highest-risk group. Due to the expected rapid and relentless progression of spinal collapse, this group requires the most rigorous and frequent radiological surveillance.

### 3.3. Imaging Standards and Quantification

There was unanimous agreement (100%, excellent consensus) that, for both the Amber and Red groups, the baseline assessment should include a posteroanterior (PA) radiograph of the entire spine and pelvis, obtained exclusively in the seated position. Additionally, the panel reached strong consensus (93%) that pelvic obliquity be defined as a vertical tilt of ≥5° between the iliac crests relative to the horizontal plane.

### 3.4. Surveillance Intervals and the Risk-Stratified Timetable

**Figure 2:**
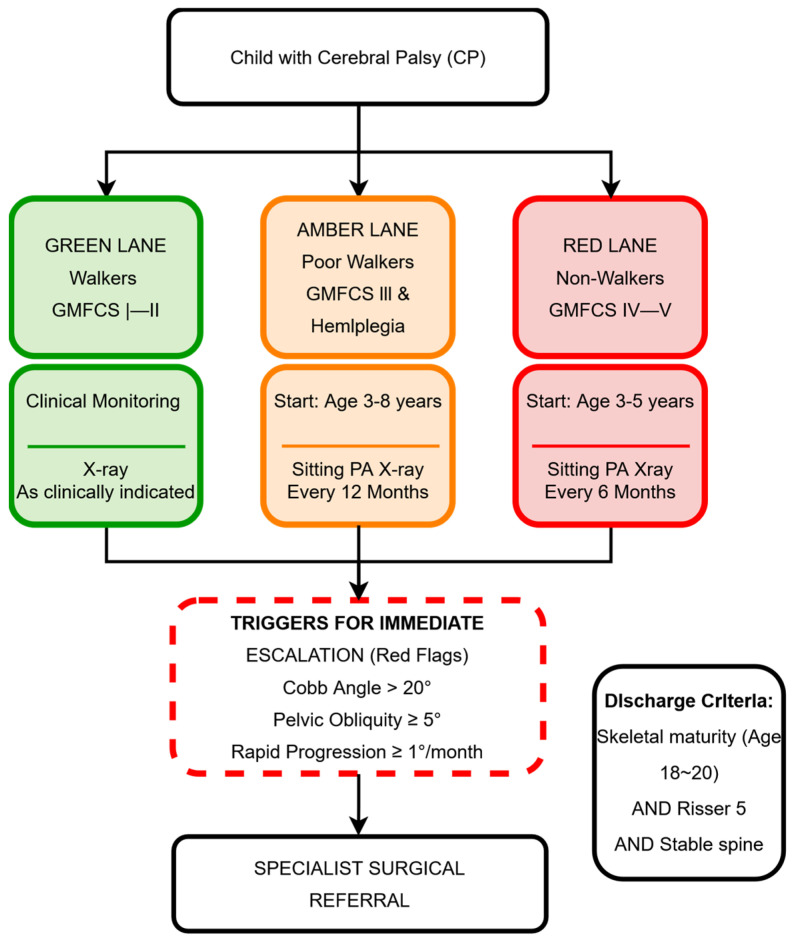
Flowchart of the consensus-based “Traffic Light” Spine Surveillance Protocol. Patients are classified into three risk groups based on functional status (GMFCS I−V). The green, yellow, and red boxes represent the low-risk, moderate-risk, and high-risk surveillance pathways, respectively. The protocol outlines a structured radiographic schedule and highlights key exit points (“Red Flags”). Pelvic obliquity (≥5°) or rapid progression (≥1°/month) triggers immediate specialist referral (indicated by the red dashed lines), regardless of the patient’s current surveillance level. Patients who have reached skeletal maturity (18−20 years) and have a stable spine are discharged.

The consensus established a specific radiographic timetable based on the established risk groups, as summarized in Figure 2.

Green Lane: Routine radiographic surveillance is not recommended (86% agreement, Excellent Consensus). Assessment depends entirely on clinical suspicion.

Amber Lane: Surveillance should begin between ages 3 and 8, with an annual radiographic review.

Red Lane: Due to the high risk of rapid deterioration, surveillance begins between ages 3 and 5. The panel reached consensus (73%) on surveillance every 6 months. This interval was chosen to reduce the risk of missing rapid, non-linear progression (around ≥1°/month) common in this group [15], which could cause a significant (>12°) deterioration that might otherwise be overlooked during a standard annual check.

### 3.5. Triggers for Escalation and Specialist Referral

The protocol clearly defines “exit points” from routine surveillance to specialist surgical evaluation. While a Cobb angle >20° remains a standard indication for referral, the panel reached excellent consensus (93%) on two independent triggers for immediate escalation, regardless of the coronal curve size: Pelvic Obliquity—vertical tilt ≥5° and Rapid Progression—deterioration of ≥1° per month.

Termination surveillance is recommended to continue until skeletal maturity, defined by a combination of chronological age (18−20 years), radiographic stability, and a Risser sign of 5.

**Figure 3:**
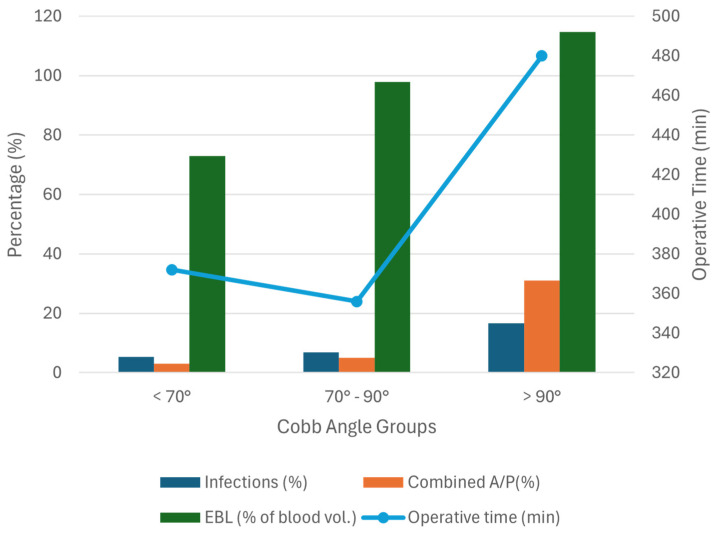
The Impact of Curve Magnitude on Surgical Complexity and Morbidity. The combo chart shows a rapid, nonlinear increase in surgical risk once the Cobb angle exceeds 90°. Entering the “Salvage Zone” (>90°) is associated with a threefold rise in infection rates, a sixfold increase in the need for high-risk anterior–posterior procedures, significantly higher estimated blood loss (EBL), and notably longer operative times compared with the “Proactive Window” (<90°). The graph was generated from data published by Hollenbeck et al. [9].

**Figure 4:**
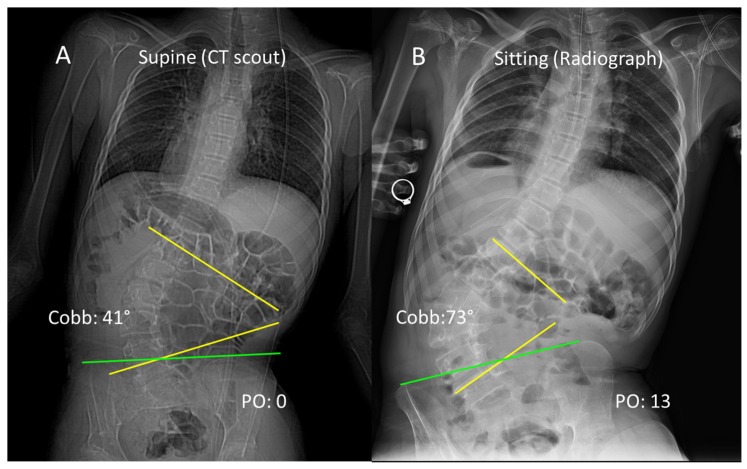
Positional Dependency of Radiographic Measurements in Non-ambulatory Patients. > Comparison of supine (**A**) and unsupported sitting (**B**) coronal imaging in a GMFCS V patient, obtained on the same day. Image (**A**) is a supine computed tomography (CT) scout view, used to minimize additional radiation exposure, and shows an artificial correction of the curvature and pelvis due to the removal of gravity. Image (**B**) is a sitting posteroanterior (PA) radiograph revealing the true functional trunk collapse and significant pelvic obliquity. This emphasizes the risk of relying solely on supine imaging, which can hide the criteria for surgical referral. Abbreviations: PO, pelvic obliquity. The yellow lines represent the measurement of the Cobb angle, and the green line indicates pelvic obliquity.

The final expert consensus also produced illustrative guidelines for radiographic evaluation and clinical presentation of the risk groups (Figure 3 and Figure 4).

## 4. Discussion

This study presents a standardized “Traffic Light” surveillance protocol for spinal deformity in children with cerebral palsy (CP), addressing a critical gap in international pediatric orthopedic care. By categorizing patients by GMFCS level, we provide a practical framework that builds on the successful design of existing hip surveillance programs and accounts for the unique biomechanical and economic challenges of neuromuscular scoliosis.

Our agreement to lower referral thresholds and maintain frequent surveillance for non-ambulatory children is based primarily on the “window of opportunity”, which closes quickly as the spinal deformity approaches a 90° Cobb angle [9]. Evidence indicates that operating before reaching this threshold greatly improves the surgery’s risk–benefit profile [19].

Once a curve exceeds 90°, surgical complexity increases disproportionately: infection rates triple from approximately 5.3–6.8% to 16.7% [9], and the need for high-risk combined anterior–posterior procedures rises sixfold from 5% to 31% [9]. Additionally, estimated blood volume loss (EBL) jumps from 72.9–97.9% to 114.7% [9]. Importantly, total operative time remains fairly stable and even slightly optimized within the “window of opportunity” (372 min for curves less than 70°, and 356 min for curves between 70° and 90°), but it increases markedly to 480 min once the 90° threshold is exceeded [9] (Figure 3).

From a functional perspective, children with moderate curves (70–90°) can achieve quality-of-life scores (CPCHILD) comparable to those of patients treated earlier. However, patients with curves exceeding 90° face a “ceiling effect” that limits their maximum potential for postoperative functional recovery and sitting balance [9].

Our protocol’s focus on 6-monthly surveillance for GMFCS IV–V children is designed to prevent patients from crossing the 90° “point of no return” during rapid pubertal growth spurts. Delaying referral until the curve becomes severe and rigid creates a vicious cycle of respiratory and infectious morbidity. Postoperative pneumonia is the most common complication in this cohort, affecting up to 43% of CP patients with predominantly GMFCS IV-V status [20]. Operating on massive curves in the “salvage zone” often prevents optimal correction of the deformity. This is critical because Levine et al. demonstrated that achieving less than 50% curve correction independently doubles the risk of postoperative pneumonia [21]. Similarly, Mohamed Ali et al. found that each 1-degree increase in the residual postoperative Cobb angle increases the risk of deep surgical site infection by 4% [22]. These findings align with the broader literature, which identifies 60–70° as the critical inflection point at which major pulmonary and general surgical complications begin to escalate significantly, further supporting our protocol’s proactive escalation triggers [20,21,22].

There are specific biomechanical reasons for including ambulant patients with asymmetric hemiplegia (GMFCS I–II) in the higher-risk ‘Amber’ pathway. While typical ‘Walkers’ (bilateral GMFCS I–II) experience relatively symmetrical axial loading, hemiplegic patients face a constant muscular imbalance. This unilateral spasticity acts as a ‘silent killer’ of spinal alignment by causing persistent pelvic obliquity; the pelvis is rarely level during gait, forcing the spine to develop compensatory curves [23]. This biomechanical challenge becomes critical during puberty. While growth rate is symmetrical in typical GMFCS I–II patients, in hemiplegic patients, the rapid increase in height, combined with a pre-existing pelvic tilt, causes the compensatory scoliosis to worsen quickly, as predicted by physiological models of curve progression [3], thereby justifying their classification alongside ‘Poor Walkers’.

The panel reached 100% consensus on the need for sitting radiographs for non-ambulatory patients. This is essential for diagnostic accuracy because spinal and pelvic parameters are highly position-dependent. Transitioning from a seated to a supine position can artificially reduce pelvic tilt by approximately 19° [14] and decrease coronal Cobb angles by 12° to 16° [13]. In neuromuscular scoliosis, lumbar lordosis often decreases significantly in the supine position due to trunk collapse and reduced active postural correction. Consequently, supine imaging in non-ambulatory children may lead to a dangerous underestimation of curve magnitude and pelvic obliquity, potentially delaying necessary surgical intervention (Figure 4).

The urgent need for a standardized surveillance protocol is underscored by the limited effectiveness of conservative management in neuromuscular scoliosis. Unlike idiopathic scoliosis, where rigid bracing can effectively halt curve progression and avoid surgery, bracing in CP is primarily supportive rather than corrective. Custom-molded seating systems and thoracolumbosacral orthoses (TLSO) are useful for enhancing postural support, improving sitting balance, and aiding upper extremity function. However, the existing literature consistently shows that orthotic management does not alter the natural course of CP scoliosis or permanently prevent curve progression [24]. Because conservative measures cannot halt spinal curve progression, early surgical referral remains the only definitive option for progressive curves. Therefore, the main purpose of the ‘Traffic Light Protocol’ is not to identify patients for bracing but to accurately detect the critical point at which surgery becomes necessary while remaining safe. Identifying a curve at 50° to 70° enables the surgical team to perform a carefully planned fusion.

Implementing the “Traffic Light” system could directly address barriers in the referral pathway. Significant knowledge gaps among primary care pediatricians regarding the diagnosis and natural history of severe neuromuscular conditions result in delayed referrals [12]. This issue is compounded by limited parental engagement in posture surveillance and geographic disparities in access to specialists [11,25]. Research shows that a family’s socioeconomic situation often changes significantly when a child requires intensive scoliosis treatment [11]. By using objective triggers—such as pelvic obliquity ≥5° or progression ≥1° per month—the protocol can reduce reliance on subjective clinical judgment. It promotes timely specialist intervention, thereby closing the gap between primary care and surgical centers. This velocity-based deterioration metric emerged explicitly from the Delphi panel discussions and is grounded in the natural history of CP scoliosis.

The economic rationale for early, standardized surveillance is compelling. Pediatric CP patients undergoing spinal fusion already incur significantly higher hospital costs—about $13,482 more per case—than non-CP patients [26]. Complications, such as readmissions, can add an additional $49,000 per episode [27]. Because salvage procedures for neglected curves greater than 90° are inherently associated with higher infection rates, longer operative times, and greater use of intensive care resources, the financial impact of delayed referral is considerable. Proactive intervention under this protocol, combined with optimized perioperative care [28], offers an effective strategy for managing healthcare costs.

While establishing the ‘Traffic Light Protocol’ can be a critical step forward, its ultimate success depends on widespread clinical implementation and future validation. Future research should prioritize prospective, multicenter cohort studies comparing clinical outcomes of patients managed under this protocol with historical cohorts lacking structured surveillance. Determining whether the protocol measurably reduces the incidence of curves >90° at the time of surgery would be the definitive test of its efficacy. Furthermore, integration with existing digital healthcare infrastructure is essential. The protocol should ideally be embedded in electronic medical records (EMR) as an automated alert system. Looking forward, rapid advances in Artificial Intelligence (AI) and machine learning in musculoskeletal imaging offer a transformative application for this protocol. AI algorithms trained to automatically calculate Cobb angles and detect pelvic obliquity on routine abdominal or chest radiographs could serve as opportunistic screening tools, further reducing the clinical burden on orthopedic clinics [29]. Finally, future iterations of these guidelines should address global health disparities. The current consensus primarily reflects expert opinion from high-resource healthcare systems. Adapting the ‘Traffic Light Protocol’ for Low- and Middle-Income Countries (LMICs)—where access to spine surgeons and routine radiography is severely limited—requires further research to determine the safest possible extended intervals [30].

Despite the rigor of the Delphi process, the 14 experts are from high-volume academic centers in Poland, the Czech Republic, Finland, and the United Kingdom, and their practices primarily reflect European patterns. As with all Delphi studies, the resulting protocol is based on expert consensus (Level V evidence) rather than on data from a randomized controlled trial. Finally, although GMFCS is the gold standard for functional classification, surgical timing in clinical practice should also take into account many patient-specific factors, including nutritional status, seizure control, and baseline pulmonary function. Furthermore, because the expert panel was predominantly composed of European specialists, the protocol may require regional adaptation for use in other healthcare settings, such as insurance-based models in North America or diverse healthcare infrastructures in Asia. 

It is crucial to emphasize that the Traffic Light Protocol can serve as a baseline framework. Patient-specific factors and severe comorbidities, such as poor nutritional status or uncontrolled seizures, should independently trigger individualized, higher-risk surveillance.

## 5. Conclusions

The “Traffic Light” protocol provides a much-needed, risk-based framework for spinal surveillance in children with cerebral palsy. By shifting the clinical approach from “reactive salvage” to “proactive preservation”, the system aims to prevent any child from reaching the 90° “point of no return” because of referral delays or inconsistent imaging protocols. Ultimately, this protocol can give the larger multidisciplinary clinical team—including primary care pediatricians, neurologists, and physiotherapists—a practical tool to prevent severe pulmonary issues and early death from neglected neuromuscular spinal deformity.

## Figures and Tables

**Table 1:** Summary of Key Consensus Statements.

Domain	Consensus Statement	Agreement (%)	Consensus Level
Classification	Stratify into: Green (Walkers), Amber (Poor Walkers), Red (Non-Walkers).	93%	Excellent
Imaging	Standard for non-ambulatory: PA full-spine + pelvis, sitting position.	100%	Excellent
Frequency	Non-Walkers (Red Group) require surveillance every 6 months.	73%	Good
Escalation	Independent triggers: Pelvic obliquity ≥5° OR Progression ≥1°/month.	93%	Excellent
Referral	Mandatory referral for Cobb >20°, pain, or functional decline.	93%	Excellent
Discharge	End surveillance at maturity (age 18–20, Risser 5) only if stable.	86%	Excellent

## Data Availability

The anonymized data supporting the consensus results of this Delphi study are available from the corresponding author upon reasonable request.

## Supplementary Materials

The following supporting information can be downloaded at: https://www.mdpi.com/article/10.3390/jcm15093205/s1. Supplementary Material S1: Table S1: The evolution of selected Delphi statements and voting outcomes across the rounds; Table S2: the qualitative dataset from Round 1; Supplementary Data S2: Detailed raw data from the expert consensus.

## Abbreviations

The following abbreviations are used in this manuscript:

AI	Artificial Intelligence
AIS	Adolescent Idiopathic Scoliosis
CP	Cerebral Palsy
CPUP	Cerebral Palsy Follow-Up Program (Cerebral Pares Uppföljnings Program)
CREDES	Conducting and Reporting Delphi Studies
EMR	Electronic Medical Records
EPOS	European Paediatric Orthopaedic Society
GMFCS	Gross Motor Function Classification System
LMICs	Low- and Middle-Income Countries
PA	Posteroanterior
TLSO	Thoracolumbosacral Orthosis

## References

[1] Himmelmann K., McIntyre S., Goldsmith S., Smithers-Sheedy H., Watson L. (2020). Epidemiology of Cerebral Palsy. Cerebral Palsy.

[2] Bertoncelli C.M., Solla F., Loughenbury P.R., Tsirikos A.I., Bertoncelli D., Rampal V. (2017). Risk Factors for Developing Scoliosis in Cerebral Palsy: A Cross-Sectional Descriptive Study. J. Child Neurol..

[3] Saito N., Ebara S., Ohotsuka K., Kumeta H., Takaoka K. (1998). Natural History of Scoliosis in Spastic Cerebral Palsy. Lancet.

[4] Terjesen T., Vinje S., Kibsgård T. (2024). The Relationship between Hip Displacement, Scoliosis, and Pelvic Obliquity in 106 Nonambulatory Children with Cerebral Palsy: A Longitudinal Retrospective Population-Based Study. Acta Orthop..

[5] Hägglund G., Pettersson K., Czuba T., Persson-Bunke M., Rodby-Bousquet E. (2018). Incidence of Scoliosis in Cerebral Palsy. Acta Orthop..

[6] Willoughby K.L., Ang S.G., Thomason P., Rutz E., Shore B., Buckland A.J., Johnson M.B., Graham H.K. (2022). Epidemiology of Scoliosis in Cerebral Palsy: A Population-Based Study at Skeletal Maturity. J. Paediatr. Child Health.

[7] Ahonen M., Jeglinsky-Kankainen I., Gissler M., Helenius I. (2025). Mortality for Pneumonia and Risk of Pneumonia in Children with Cerebral Palsy Treated with and without Surgery. Eur. Spine J..

[8] Hägglund G., Alriksson-Schmidt A., Lauge-Pedersen H., Rodby-Bousquet E., Wagner P., Westbom L. (2014). Prevention of Dislocation of the Hip in Children with Cerebral Palsy: 20-Year Results of a Population-Based Prevention Programme. Bone Jt. J..

[9] Hollenbeck S.M., Yaszay B., Sponseller P.D., Bartley C.E., Shah S.A., Asghar J., Abel M.F., Miyanji F., Newton P.O. (2019). The Pros and Cons of Operating Early Versus Late in the Progression of Cerebral Palsy Scoliosis. Spine Deform..

[10] Chhabra B., Birhiray D., Deveza L., Gremillion M., McHorse G., Dahl B., Gerow F., Hanson D., Smith B. (2025). Does a Delay of Surgery Due to a Multidisciplinary Screening Process Result in Neuromuscular Scoliosis Curve Progression in Complex Cerebral Palsy?. Int. Orthop..

[11] Latalski M., Fatyga M., Kuzaka R., Bylina J., Trzpis T., Kopytiuk R., Jarosz M.J., Latalska M. (2012). Socio-Economic Conditionings of Families with Children Treated Due to Scoliosis in Eastern Poland. Ann. Agric. Environ. Med..

[12] Beisenbaeva G.G., Ryskulova A.R., Izbasarova A.S., Nurlybaeva G.A., Zhumadilova A.N. (2024). Analysis of physicians’ knowledge on the diagnosis and treatment of spinal muscular atrophy using a developed validated questionnaire. Nauchno-Prakt. Zhurnal Ftiziopul’monologiya.

[13] Ramirez N., Padilla J., Villarin S., Irizarry F., Iriarte I., Sawyer J. (2019). Impact of Patient Position on Coronal Cobb Angle Measurement in Non-Ambulatory Myelodysplastic Patients. Eur. J. Orthop. Surg. Traumatol..

[14] Durbas A., Subramanian T., Simon C., Allen M.R.J., Samuel J., Colón L.F., Mazzucco M.R., Pagan C., Karasavvidis T., Vigdorchik J. (2025). Evaluating Variations in Spinopelvic Parameters from Sitting to Standing: A Comparative Analysis of 1447 Older Adults Across Age, BMI, and Gender Subgroups. J. Clin. Med..

[15] Niederberger M., Spranger J. (2020). Delphi Technique in Health Sciences: A Map. Front. Public Health.

[16] Hasson F., Keeney S. (2011). Enhancing Rigour in the Delphi Technique Research. Technol. Forecast. Soc. Change.

[17] Jünger S., Payne S.A., Brine J., Radbruch L., Brearley S.G. (2017). Guidance on Conducting and REporting DElphi Studies (CREDES) in Palliative Care: Recommendations Based on a Methodological Systematic Review. Palliat. Med..

[18] Ilharreborde B., Yazici M., Yüksel S., Demir P., Helenius I. (2025). Early Onset Scoliosis: Can Best Practice Guidelines Be Provided in Europe?. J. Child. Orthop..

[19] Miyanji F., Nasto L.A., Sponseller P.D., Shah S.A., Samdani A.F., Lonner B., Yaszay B., Clements D.H., Narayanan U., Newton P.O. (2018). Assessing the Risk-Benefit Ratio of Scoliosis Surgery in Cerebral Palsy: Surgery Is Worth It. J. Bone Joint Surg. Am..

[20] Vandendriessche E., Proesmans M., Ortibus E., Moens P. (2021). Complication Rate after Scoliosis Surgery in Children with Cerebral Palsy. Acta Orthop. Belg..

[21] Levine S.B., Fields M.W., Boby A.Z., Matsumoto H., Skaggs K.F., Roye B.D., Vitale M.G. (2022). Degree of Postoperative Curve Correction Decreases Risks of Postoperative Pneumonia in Patients Undergoing Both Fusion and Growth-Friendly Surgical Treatment of Neuromuscular Scoliosis. J. Pediatr. Orthop..

[22] Mohamed Ali M.H., Koutharawu D.N., Miller F., Dabney K., Gabos P., Shah S., Holmes L. (2010). Operative and Clinical Markers of Deep Wound Infection after Spine Fusion in Children with Cerebral Palsy. J. Pediatr. Orthop..

[23] Hägglund G., Goldring M., Hermanson M., Rodby-Bousquet E. (2018). Pelvic Obliquity and Measurement of Hip Displacement in Children with Cerebral Palsy. Acta Orthop..

[24] Merkelbach N., Pauw A.D., Van Campenhout A. (2025). Bracing for Scoliosis in Children with Cerebral Palsy—A Systematic Review. J. Child. Orthop..

[25] Clark S. (2008). Waiting Times for Scoliosis Surgery. Lancet.

[26] Anastasio A.T., Guisse N.F., Farley K.X., Rhee J.M. (2022). Hospital Burdens of Patients with Cerebral Palsy Undergoing Posterior Spinal Fusion for Scoliosis. Glob. Spine J..

[27] Lee N.J., Fields M., McCormick K.L., Hong D., Kim J.S., Lombardi J.M., Roye B.D., Lenke L.G. (2020). The Morbidity, Readmissions, and Cost for Pediatric Cerebral Palsy Patients Undergoing Primary Spinal Fusion Surgery: A National Analysis of 2779 Patients. Spine J..

[28] Naume M.M., Hoei-Hansen C.E., Born A.P., Brekke G., Høj A., Nielsen M.R., Borgwardt L., Vissing J., Dirks J., Rye A.K.S. (2024). A Prospective Study on the Feasibility and Effect of an Optimized Perioperative Care Protocol in Pediatric Neuromuscular Scoliosis Surgery. J. Clin. Med..

[29] Li H., Qian C., Yan W., Fu D., Zheng Y., Zhang Z., Meng J., Wang D. (2024). Use of Artificial Intelligence in Cobb Angle Measurement for Scoliosis: Retrospective Reliability and Accuracy Study of a Mobile App. J. Med. Internet Res..

[30] Monteiro F.N.S., Alexandre M., Santos W.Z., Mendonça R.G.M.D., Gotfryd A.O., Caffaro M.F.S., Meves R. (2023). Study on pediatric scoliosis patients at Hospital Santa Casa de Misericórdia in São Paulo. Coluna/Columna.

