# Supplementary material for: The Traffic Light Protocol: Preventing the 90° ‘Point of No Return’ Through Risk-Stratified Spinal Surveillance in Children with Cerebral Palsy
Source: J Clin Med. 2026 Apr 22;15(9):3205. doi: 10.3390/jcm15093205 (PMC13164426; doi:10.3390/jcm15093205)
Supplement: Supplementary file 1 [file jcm-15-03205-s001.zip › Supplementary Material S1.pdf]

Supplementary Material S1

Title: The Traffic Light Protocol: Preventing the 90° 'Point of No Return' through Risk-Stratified Spinal Surveillance in Children with Cerebral Palsy **Authors:** Michał Latalski, et al.

Table S1: Evolution of Selected Delphi Statements and Voting Outcomes Across Rounds

Note: To ensure transparency, this table summarizes key statements evaluated across the Delphi process, including items that were modified or failed to reach the predefined consensus thresholds (Excellent ≥80%, Good ≥73%).

Delphi Round	Evaluated Statement / Clinical Variable	Agreement (%)	Consensus Level	Action Taken / Outcome
**Round 1**	Routine radiographic spinal surveillance is mandatory for all ambulatory patients (GMFCS I-II).	46%	No Consensus	**Rejected.** Modified in Round 2 to focus clinical monitoring on symmetric GMFCS I-II patients (Green Group).
**Round 1**	Age is the primary determinant for the frequency of spinal surveillance in CP.	53%	No Consensus	**Rejected.** Modified to recognize GMFCS level (motor function) as the primary determinant.
**Round 1**	Standing radiographs are adequate for non-ambulatory patients if supported by caregivers.	13%	No Consensus	**Rejected.** Rephrased to explicitly mandate sitting, unsupported (or minimally supported) radiographs to unmask pelvic obliquity.
**Round 2**	Patients in the Amber Group (GMFCS III and asymmetric hemiplegia) require radiographs every 6 months.	58%	No Consensus	**Modified.** Interval extended; Round 3 tested annual (12-month) frequency for this specific group.
**Round 2**	Patients in the Red Group (GMFCS IV-V) require radiographic evaluation every 12 months.	40%	No Consensus	**Rejected.** Interval deemed too long due to the risk of rapid progression. Modified to 6-monthly evaluations.
**Round 2**	An isolated coronal Cobb angle >20° is the sole trigger for surgical referral.	66%	No Consensus	**Modified.** Expanded to include pelvic obliquity and progression velocity as independent escalation triggers.

**Round 3**	Amber Group (Poor Walkers) requires annual (every 12 months) radiographic surveillance starting between ages 3–8.	86%	Excellent	**Accepted.** Included in the final Traffic Light Protocol.
**Round 3**	Red Group (Non-Walkers) requires 6-monthly radiographic surveillance starting between ages 3–5.	73%	Good	**Accepted.** Included in the final Traffic Light Protocol.
**Round 3**	Pelvic obliquity of ≥5° serves as an independent "Red Flag" trigger for immediate specialist referral.	93%	Excellent	**Accepted.** Included in the final Traffic Light Protocol.
**Round 3**	Rapid curve progression of ≥1° per month serves as an independent "Red Flag" trigger for referral.	93%	Excellent	**Accepted.** Included in the final Traffic Light Protocol.
**Round 3**	Spinal surveillance can be safely terminated at skeletal maturity (Age 18-20, Risser 5) if the spine is clinically and radiographically stable.	86%	Excellent	**Accepted.** Included in the final discharge criteria.

Table S2.1 below presents the full qualitative dataset from the initial exploratory phase (Round 1). The complete quantitative raw datasets from Round 2 (135 items) and Round 3 (35 items) are provided as a separate Excel file in Supplementary Data S2 to ensure full transparency and open science standards

**Delphi Round**	**Evaluated Statement / Clinical Variable**	**Agreement (%)**	**Consensus Level**	**Action Taken / Outcome**
**Round 1**	Routine radiographic spinal surveillance is mandatory for all ambulatory patients (GMFCS I-II).	46%	No Consensus	**Rejected.** Modified in Round 2 to focus clinical monitoring on symmetric GMFCS I-II patients (Green Group).
**Round 1**	Age is the primary determinant for the frequency of spinal surveillance in CP.	53%	No Consensus	**Rejected.** Modified to recognize GMFCS level (motor function) as the primary determinant.
**Round 1**	Standing radiographs are adequate for non-ambulatory patients if supported by caregivers.	13%	No Consensus	**Rejected.** Rephrased to explicitly mandate sitting, unsupported (or minimally supported)

				radiographs to unmask pelvic obliquity.
**Round 2**	Patients in the Amber Group (GMFCS III and asymmetric hemiplegia) require radiographs every 6 months.	58%	No Consensus	**Modified.** Interval extended; Round 3 tested annual (12-month) frequency for this specific group.
**Round 2**	Patients in the Red Group (GMFCS IV-V) require radiographic evaluation every 12 months.	40%	No Consensus	**Rejected.** Interval deemed too long due to the risk of rapid progression. Modified to 6-monthly evaluations.
**Round 2**	An isolated coronal Cobb angle >20° is the sole trigger for surgical referral.	66%	No Consensus	**Modified.** Expanded to include pelvic obliquity and progression velocity as independent escalation triggers.
**Round 3**	Amber Group (Poor Walkers) requires annual (every 12 months) radiographic surveillance starting between ages 3–8.	86%	Excellent	**Accepted.** Included in the final Traffic Light Protocol.
**Round 3**	Red Group (Non-Walkers) requires 6-monthly radiographic surveillance starting between ages 3–5.	73%	Good	**Accepted.** Included in the final Traffic Light Protocol.
**Round 3**	Pelvic obliquity of ≥5° serves as an independent "Red Flag" trigger for immediate specialist referral.	93%	Excellent	**Accepted.** Included in the final Traffic Light Protocol.
**Round 3**	Rapid curve progression of ≥1° per month serves as an independent "Red Flag" trigger for referral.	93%	Excellent	**Accepted.** Included in the final Traffic Light Protocol.
**Round 3**	Spinal surveillance can be safely terminated at skeletal maturity (Age 18-20, Risser 5) if the spine is clinically and radiographically stable.	86%	Excellent	**Accepted.** Included in the final discharge criteria.
